# Supplementary material for: Residual Platelet Reactivity and Dyslipidemia in Post-CABG Patients Undergoing Repeat Revascularization: Insights from Kazakhstan
Source: Diseases. 2025 Nov 9;13(11):365. doi: 10.3390/diseases13110365 (PMC12651223; doi:10.3390/diseases13110365)
Supplement: Supplementary file 1 [file diseases-13-00365-s001.zip › diseases-3919843-supplementary.pdf]

Supplementary Materials

Table S1. Full study: Descriptive statistics of the group without and with re-stenting.

	**Without re-stenting**	**With re-stenting**	**p-value**	**N**
	N=59	***N=136***		
Age	66.0 [61.5;73.0]	71.0 [65.8;75.0]	0.016	195
Gender:			0.431	195
female	13 (22.0%)	39 (28.7%)		
male	46 (78.0%)	97 (71.3%)		
Smoking:			0.001	195
No	27 (45.8%)	97 (71.3%)		
Yes	32 (54.2%)	39 (28.7%)		
Alcohol:			0.164	195
No	56 (94.9%)	134 (98.5%)		
Yes	3 (5.08%)	2 (1.47%)		
Weight	79.0 [70.0;86.5]	78.5 [70.0;85.8]	0.903	195
Height	170 [164;174]	168 [162;173]	0.438	195
BMI	27.3 [24.9;29.4]	27.6 [25.0;30.2]	0.411	195
Heredity:			0.874	195
No	37 (62.7%)	82 (60.3%)		
Yes	22 (37.3%)	54 (39.7%)		
Dyspnea:			0.776	194
No	5 (8.47%)	10 (7.41%)		
Yes	54 (91.5%)	125 (92.6%)		
Edema:			0.190	195
No	55 (93.2%)	116 (85.3%)		
Yes	4 (6.78%)	20 (14.7%)		
Pain:			0.726	195
No	2 (3.39%)	7 (5.15%)		
Yes	57 (96.6%) ?	129 (94.9%)		
Weakness:			0.104	195
No	0 (0.00%)	7 (5.15%)		
Yes	59 (100%)	129 (94.9%)		
Heartbeat:			0.451	195
No	53 (89.8%)	115 (84.6%)		
Yes	6 (10.2%)	21 (15.4%)		
Short of breath:			0.961	195
No	43 (72.9%)	97 (71.3%)		
Yes	16 (27.1%)	39 (28.7%)		
SBP	120 [110;130]	130 [120;140]	0.041	195
DBP	80.0 [70.0;80.0]	80.0 [70.0;80.0]	0.201	195
History of myocardial infarction:			0.145	195
No	33 (55.9%)	59 (43.4%)		
Yes	26 (44.1%)	77 (56.6%)		
Arterial hypertension:			0.133	195
No	5 (8.47%)	4 (2.94%)		
Yes	54 (91.5%)	132 (97.1%)		

	Without re-stenting	With re-stenting	p-value	N
	N=59	*N=136*		
DM type 2:			0.603	195
No	42 (71.2%)	90 (66.2%)		
Yes	17 (28.8%)	46 (33.8%)		
History of Stroke:			0.400	195
No	56 (94.9%)	123 (90.4%)		
Yes	3 (5.08%)	13 (9.56%)		
CKD:			0.068	195
No	39 (66.1%)	69 (50.7%)		
Yes	20 (33.9%)	67 (49.3%)		
Stenting:			0.712	195
No	47 (79.7%)	113 (83.1%)		
Yes	12 (20.3%)	23 (16.9%)		
CABG:			0.003	195
No stents before CARB	10 (16.9%)	5 (3.68%)		
Yes – there were stents before CABG	49 (83.1%)	131 (96.3%)		
IHD, duration	3.00 [1.00;6.50]	2.50 [1.00;10.0]	0.364	195
Hemoglobin	142 [132;154]	145 [135;155]	0.542	195
CRP	2.00 [1.24;3.56]	2.45 [1.03;4.22]	0.487	195
Glucose	5.54 [5.12;6.30]	5.80 [5.16;6.93]	0.293	195
HbA1c	6.01 [5.30;6.70]	6.05 [5.40;7.33]	0.403	195
Creatinine	80.4 [70.6;90.2]	79.0 [70.8;92.2]	0.952	195
GFR	88.0 [75.8;96.0]	81.0 [69.8;94.2]	0.279	195
Potassium	4.40 [4.20;4.60]	4.30 [4.10;4.60]	0.590	195
Sodium	142 [140;144]	142 [141;144]	0.268	195
ALT	19.0 [14.1;28.5]	18.1 [12.9;25.7]	0.453	195
AST	17.3 [13.8;25.9]	17.9 [14.9;23.1]	0.977	195
TC	4.24 [3.41;5.25]	4.50 [3.70;5.22]	0.419	195
LDL	2.58 [2.00;3.17]	2.42 [2.00;3.20]	0.569	195
HDL	1.00 [0.99;1.00]	1.00 [0.96;1.03]	0.809	195
TG	1.50 [1.00;1.98]	1.29 [1.00;1.75]	0.083	195
Troponin I	20.0 [13.4;95.4]	14.3 [10.3;32.8]	0.002	195
TSH	2.00 [1.47;2.90]	2.00 [1.39;3.15]	0.773	195
Thyroxine	14.1 [12.0;16.0]	14.0 [11.6;16.2]	0.945	193
PTI	94.9 [80.3;103]	95.8 [77.5;105]	0.627	195
INR	1.07 [1.04;1.17]	1.04 [0.99;1.14]	0.039	195
APTT	31.1 [28.8;36.5]	31.6 [28.7;35.7]	0.737	195
EDV	162 [129;206]	136 [113;179]	0.038	195
ESV	80.0 [45.5;116]	61.5 [45.8;95.2]	0.222	195
SV	78.0 [69.5;87.5]	77.5 [59.8;92.2]	0.466	195
sPAP	32.0 [23.0;44.0]	35.0 [24.5;42.0]	0.917	194
EF	47.0 [41.5;59.0]	52.0 [44.0;59.2]	0.235	195
LA	3.80 [3.60;4.55]	3.90 [3.50;4.40]	0.638	195
EDD	5.70 [5.20;6.30]	5.30 [4.97;6.00]	0.081	195
ESD	4.20 [3.35;4.90]	3.80 [3.30;4.60]	0.240	195
LVPW	1.05 [0.90;1.20]	1.10 [1.00;1.20]	0.030	195
IVS	1.10 [0.95;1.27]	1.10 [1.00;1.30]	0.924	195
MV:			0.858	195
No	4 (6.78%)	13 (9.56%)		

	Without re-stenting	With re-stenting	p-value	N
	N=59	*N=136*		
1 degree.	36 (61.0%)	75 (55.1%)		
2 degree.	13 (22.0%)	36 (26.5%)		
3 degree.	6 (10.2%)	11 (8.09%)		
4 degree.	0 (0.00%)	1 (0.74%)		
TV:			0.437	195
No	9 (15.3%)	22 (16.2%)		
1 degree.	32 (54.2%)	67 (49.3%)		
2 degree.	16 (27.1%)	46 (33.8%)		
3 degree.	2 (3.39%)	1 (0.74%)		
AV:			0.309	195
No	30 (50.8%)	71 (52.2%)		
1 degree.	22 (37.3%)	49 (36.0%)		
2 degree.	5 (8.47%)	15 (11.0%)		
3 degree.	0 (0.00%)	1 (0.74%)		
Stenosis	2 (3.39%)	0 (0.00%)		
RV	3.00 [2.80;3.40]	3.10 [2.90;3.40]	0.566	195
LV aneurysm:			0.990	195
No	50 (84.7%)	117 (86.0%)		
Yes	9 (15.3%)	19 (14.0%)		
Heart Rate	75.0 [66.0;82.0]	71.5 [65.0;83.2]	0.496	195
USD of BCA:			1.000	195
No	24 (40.7%)	56 (41.2%)		
Yes	35 (59.3%)	80 (58.8%)		
RCA, degree:			0.117	195
minor	44 (74.6%)	84 (61.8%)		
significant	15 (25.4%)	52 (38.2%)		
LAD, degree:			0.002	195
Minor	43 (72.9%)	64 (47.1%)		
Significant	16 (27.1%)	72 (52.9%)		
LCA, degree:			0.235	195
Minor	55 (93.2%)	117 (86.0%)		
Significant	4 (6.78%)	19 (14.0%)		
CA, degree:			0.002	195
Minor	51 (86.4%)	85 (62.5%)		
significant	8 (13.6%)	51 (37.5%)		
DB, degree:			0.001	195
Minor	54 (91.5%)	94 (69.1%)		
significant	5 (8.47%)	42 (30.9%)		
PIVB, degree:			0.006	195
Minor	58 (98.3%)	113 (83.1%)		
Significant	1 (1.69%)	23 (16.9%)		
IA, degree:			0.369	195
Minor	56 (94.9%)	133 (97.8%)		
significant	3 (5.08%)	3 (2.21%)		
OMB, degree:			0.002	195
Minor	56 (94.9%)	102 (75.0%)		
Significant	3 (5.08%)	34 (25.0%)		
Number of shunts	2.00 [2.00;3.00]	3.00 [2.00;3.00]	<0.001	195

	Without re-stenting	With re-stenting	p-value	N
	N=59	*N=136*		
Number of affected vessels	3.00 [2.00;3.00]	4.00 [3.75;5.00]	<0.001	195
Statins (high-intensity statins atorvastatin 40 mg or rosuvastatin 20 mg): Yes	59 (100%)	136 (100%)	.	195
Ezetimibe:			<0.001	195
No	59 (100%)	89 (65.4%)		
Yes	0 (0.00%)	47 (34.6%)		
Angina after CABG:			<0.001	195
No	7 (11.9%)	0 (0.00%)		
Yes	52 (88.1%)	136 (100%)		
Mortality after CABG:			1.000	195
No	58 (98.3%)	132 (97.1%)		
Yes	1 (1.69%)	4 (2.94%)		
Stenting after CABG:			<0.001	195
No	59 (100%)	0 (0.00%)		
Yes	0 (0.00%)	136 (100%)		
PRU	145 [140;155]	230 [200;274]	<0.001	195
Dyslipidemia:			1.000	195
No	2 (3.39%)	4 (2.94%)		
Yes	57 (96.6%)	132 (97.1%)		
Anemia:			0.941	195
No	52 (88.1%)	122 (89.7%)		
Yes	7 (11.9%)	14 (10.3%)		
CKD:			0.977	195
No	53 (89.8%)	124 (91.2%)		
Yes	6 (10.2%)	12 (8.82%)		
Obesity:			0.406	195
No	17 (28.8%)	30 (22.1%)		
Yes	42 (71.2%)	106 (77.9%)		
RPR:			<0.001	195
Therapeutic window	59 (100%)	46 (33.8%)		
HRPR	0 (0.00%)	90 (66.2%)		

Table S2. Full study: Descriptive statistics of the Therapeutic window/HRPR group.

	Therapeutic window	HRPR	p-value	N
	N=105	*N=90*		
Age	69.0 [62.0;74.0]	70.5 [65.2;75.0]	0.147	195
Gender:			0.871	195
female	29 (27.6%)	23 (25.6%)		
male	76 (72.4%)	67 (74.4%)		
Smoking:			0.030	195
No	59 (56.2%)	65 (72.2%)		
Yes	46 (43.8%)	25 (27.8%)		
Alcohol:			1.000	195
No	102 (97.1%)	88 (97.8%)		
Yes	3 (2.86%)	2 (2.22%)		
Weight	78.0 [69.0;85.0]	80.0 [70.2;88.0]	0.199	195
Height	168 [163;174]	170 [162;173]	0.838	195

	Therapeutic window	HRPR	p-value	N
	N=105	*N=90*		
BMI	27.2 [25.0;29.4]	28.1 [25.9;30.6]	0.096	195
Heredity:			0.475	195
No	67 (63.8%)	52 (57.8%)		
Yes	38 (36.2%)	38 (42.2%)		
Dyspnea:			0.739	194
No	7 (6.67%)	8 (8.99%)		
Yes	98 (93.3%)	81 (91.0%)		
Edema:			0.801	195
No	91 (86.7%)	80 (88.9%)		
Yes	14 (13.3%)	10 (11.1%)		
Pain:			0.510	195
No	6 (5.71%)	3 (3.33%)		
Yes	99 (94.3%)	87 (96.7%)		
Weakness:			0.252	195
No	2 (1.90%)	5 (5.56%)		
Yes	103 (98.1%)	85 (94.4%)		
Heartbeat:			1.000	195
No	90 (85.7%)	78 (86.7%)		
Yes	15 (14.3%)	12 (13.3%)		
Short of breath:			0.778	195
No	74 (70.5%)	66 (73.3%)		
Yes	31 (29.5%)	24 (26.7%)		
SBP	120 [115;130]	130 [120;140]	0.031	195
DBP	80.0 [70.0;80.0]	80.0 [70.0;80.0]	0.320	195
History of myocardial infarction:			0.394	195
No	53 (50.5%)	39 (43.3%)		
Yes	52 (49.5%)	51 (56.7%)		
Arterial hypertension:			0.510	195
No	6 (5.71%)	3 (3.33%)		
Yes	99 (94.3%)	87 (96.7%)		
DM type 2:			0.293	195
No	75 (71.4%)	57 (63.3%)		
Yes	30 (28.6%)	33 (36.7%)		
History of Stroke:			0.031	195
No	101 (96.2%)	78 (86.7%)		
Yes	4 (3.81%)	12 (13.3%)		
CKD:			0.697	195
No	60 (57.1%)	48 (53.3%)		
Yes	45 (42.9%)	42 (46.7%)		
Stenting:			0.897	195
No	87 (82.9%)	73 (81.1%)		
Yes	18 (17.1%)	17 (18.9%)		
CABG:			0.065	195
No stents before CARB	12 (11.4%)	3 (3.33%)		
Yes – there were stents before CABG	93 (88.6%)	87 (96.7%)		
IHD, duration	2.00 [1.00;8.00]	3.00 [1.00;9.75]	0.888	195
Hemoglobin	142 [132;154]	150 [136;156]	0.053	195
CRP	2.36 [1.27;4.10]	2.29 [1.02;4.13]	0.853	195

	Therapeutic window	HRPR	p-value	N
	N=105	*N=90*		
Glucose	5.54 [5.00;6.20]	5.90 [5.23;7.10]	0.022	195
HbA1c	6.01 [5.38;6.70]	6.06 [5.52;7.83]	0.282	195
Creatinine	80.0 [71.0;91.8]	79.0 [70.3;92.7]	0.890	195
GFR	87.0 [74.0;96.0]	81.0 [70.0;92.5]	0.361	195
Potassium	4.30 [4.10;4.50]	4.35 [4.10;4.60]	0.267	195
Sodium	142 [141;144]	142 [140;144]	0.931	195
ALT	18.0 [12.0;27.5]	19.0 [14.0;26.4]	0.473	195
AST	17.6 [13.8;25.8]	17.7 [15.0;22.7]	0.934	195
TC	4.30 [3.67;5.27]	4.50 [3.63;5.20]	0.571	195
LDL	2.43 [2.00;3.10]	2.64 [2.00;3.39]	0.546	195
HDL	1.00 [0.98;1.01]	1.00 [0.96;1.04]	0.684	195
TG	1.37 [1.00;1.80]	1.38 [1.00;1.79]	0.981	195
Troponin I	15.0 [11.0;58.7]	15.0 [11.0;40.0]	0.635	195
TSH	2.00 [1.60;3.00]	1.96 [1.25;3.18]	0.517	195
Thyroxine	13.9 [11.5;16.2]	14.7 [12.3;16.3]	0.335	193
PTI	94.5 [80.0;103]	96.3 [78.5;106]	0.209	195
INR	1.06 [1.01;1.16]	1.04 [1.00;1.15]	0.189	195
APTT	31.4 [29.0;35.9]	31.4 [28.6;36.0]	0.524	195
EDV	157 [119;194]	131 [113;174]	0.053	195
ESV	72.0 [44.0;108]	60.0 [46.0;91.2]	0.256	195
SV	78.0 [65.0;91.0]	77.0 [59.0;90.0]	0.268	195
sPAP	34.0 [23.8;44.0]	35.0 [20.8;40.8]	0.624	194
EF	49.0 [41.0;60.0]	51.7 [45.0;58.9]	0.459	195
LA	3.80 [3.50;4.40]	3.80 [3.60;4.40]	0.907	195
EDD	5.60 [5.10;6.20]	5.20 [4.90;5.90]	0.043	195
ESD	4.00 [3.30;4.80]	3.74 [3.40;4.38]	0.394	195
LVPW	1.10 [0.90;1.20]	1.10 [1.00;1.20]	0.087	195
IVS	1.10 [1.00;1.30]	1.10 [0.90;1.30]	0.366	195
MV:			0.291	195
No	7 (6.67%)	10 (11.1%)		
1 degree.	62 (59.0%)	49 (54.4%)		
2 degree.	24 (22.9%)	25 (27.8%)		
3 degree.	12 (11.4%)	5 (5.56%)		
4 degree.	0 (0.00%)	1 (1.11%)		
TV:			0.717	195
No	14 (13.3%)	17 (18.9%)		
1 degree.	55 (52.4%)	44 (48.9%)		
2 degree.	34 (32.4%)	28 (31.1%)		
3 degree.	2 (1.90%)	1 (1.11%)		
AV:			0.070	195
No	51 (48.6%)	50 (55.6%)		
1 degree.	44 (41.9%)	27 (30.0%)		
2 degree.	7 (6.67%)	13 (14.4%)		
3 degree.	1 (0.95%)	0 (0.00%)		
Stenosis	2 (1.90%)	0 (0.00%)		
RV	3.00 [2.90;3.40]	3.10 [2.90;3.30]	0.893	195
LV aneurysm:			0.862	195
No	89 (84.8%)	78 (86.7%)		
Yes	16 (15.2%)	12 (13.3%)		

	Therapeutic window	HRPR	p-value	N
	N=105	***N=90***		
Heart Rate	74.0 [66.0;83.0]	70.5 [65.0;82.0]	0.483	195
USD of BCA:			0.196	195
No	48 (45.7%)	32 (35.6%)		
Yes	57 (54.3%)	58 (64.4%)		
RCA, degree:			0.022	195
minor	77 (73.3%)	51 (56.7%)		
significant	28 (26.7%)	39 (43.3%)		
LAD, degree:			0.002	195
Minor	69 (65.7%)	38 (42.2%)		
Significant	36 (34.3%)	52 (57.8%)		
LCA, degree:			0.694	195
Minor	94 (89.5%)	78 (86.7%)		
Significant	11 (10.5%)	12 (13.3%)		
CA, degree:			0.001	195
Minor	84 (80.0%)	52 (57.8%)		
significant	21 (20.0%)	38 (42.2%)		
DB, degree:			0.003	195
Minor	89 (84.8%)	59 (65.6%)		
significant	16 (15.2%)	31 (34.4%)		
PIVB, degree:			0.001	195
Minor	100 (95.2%)	71 (78.9%)		
Significant	5 (4.76%)	19 (21.1%)		
IA, degree:			0.688	195
Minor	101 (96.2%)	88 (97.8%)		
significant	4 (3.81%)	2 (2.22%)		
OMB, degree:			<0.001	195
Minor	98 (93.3%)	60 (66.7%)		
Significant	7 (6.67%)	30 (33.3%)		
Number of shunts	2.00 [2.00;3.00]	3.00 [2.00;4.00]	0.001	195
Number of affected vessels	3.00 [2.00;3.00]	4.00 [4.00;5.00]	<0.001	195
Statins (high-intensity statins atorvastatin 40 mg or rosuvastatin 20 mg): Yes	105 (100%)	90 (100%)	.	195
Ezetimibe:			<0.001	195
No	101 (96.2%)	47 (52.2%)		
Yes	4 (3.81%)	**43 (47.8%)**		
Angina after CABG:			0.016	195
No	7 (6.67%)	0 (0.00%)		
Yes	98 (93.3%)	90 (100%)		
Mortality after CABG:			0.183	195
No	104 (99.0%)	86 (95.6%)		
Yes	1 (0.95%)	4 (4.44%)		
Stenting after CABG:			<0.001	195
No	59 (56.2%)	0 (0.00%)		
Yes	46 (43.8%)	**90 (100%) !!!**		
PRU	170 [141;200]	255 [230;280]	<0.001	195
Dyslipidemia:			0.688	195
No	4 (3.81%)	2 (2.22%)		

	**Therapeutic window**	**HRPR**	**p-value**	**N**
	N=105	***N=90***		
Yes	101 (96.2%)	88 (97.8%)		
Anemia:			0.310	195
No	91 (86.7%)	83 (92.2%)		
Yes	14 (13.3%)	7 (7.78%)		
CKD:			0.689	195
No	94 (89.5%)	83 (92.2%)		
Yes	11 (10.5%)	7 (7.78%)		
Obesity:			0.462	195
No	28 (26.7%)	19 (21.1%)		
Yes	77 (73.3%)	71 (78.9%)		
RPR:			<0.001	195
Therapeutic window	105 (100%)	0 (0.00%)		
HRPR	0 (0.00%)	90 (100%)		
